# The role of endoscopy in the management of gastroesophageal reflux disease

**DOI:** 10.1002/deo2.86

**Published:** 2021-12-30

**Authors:** Shiko Kuribayashi, Hiroko Hosaka, Fumihiko Nakamura, Ko Nakata, Keigo Sato, Yuki Itoi, Yu Hashimoto, Kengo Kasuga, Hirohito Tanaka, Toshio Uraoka

**Affiliations:** ^1^ Department of Gastroenterology and Hepatology Gunma University Graduate School of Medicine Gunma Japan

**Keywords:** endoscopic treatment, endoscopy, gastroesophageal reflux disease

## Abstract

Gastroesophageal reflux disease (GERD) is a common disease that may cause a huge economic burden. Endoscopy is performed not only to rule out other organic diseases but also to diagnose reflux esophagitis or Barrett's esophagus. Non‐erosive GERD (non‐erosive reflux disease [NERD]) is called endoscopy‐negative GERD; however, GERD‐related findings could be obtained through histological assessment, image‐enhanced endoscopy, and new endoscopic modalities in patients with NERD. Moreover, endoscopy is useful to stratify the risk for the development of GERD. In addition, endoscopic treatments have been developed. These techniques could significantly improve patients’ quality of life as well as symptoms.

## INTRODUCTION

Gastroesophageal reflux disease (GERD) is a condition in which the refluxate of gastric content causes complications or troublesome symptoms, such as heartburn or regurgitation.^1^ GERD is one of the most common upper gastrointestinal diseases. The prevalence of GERD varies among countries, and it is estimated as 18.1%–27.8% in North America, 8.8%–25.9% in Europe, 2.5%–7.8% in East Asia.^2^ A recent systematic review based on the United Nation's 2017 Revision of World Population Prospects shows that the global prevalence of GERD is 13.98%, and the estimated number of patients who suffer from GERD is 1.03 billion.^3^ Although the prevalence of GERD is lower in East Asia than in Western countries, it may be increasing in Japan.^4^ GERD significantly decreases patients’ quality of life,^5^ and it is related to a huge economic burden.^6^

GERD is diagnosed based on the presence of reflux‐related symptoms as well as an endoscopic assessment. GERD is divided into three phenotypes: reflux esophagitis (RE), non‐erosive reflux disease (NERD), and Barrett's esophagus (BE). Mucosal breaks in endoscopy are seen in RE, while they are not seen (endoscopically negative) in NERD, though patients may feel reflux‐related symptoms. BE is characterized by columnar epithelium replacement in the distal esophagus.

It is important to exclude organic diseases by endoscopy, such as esophageal cancer, candidiasis, or eosinophilic esophagitis in patients with reflux‐related symptoms. A biopsy can be taken during endoscopy, and histological assessment can provide additional information for diagnosing GERD.

Endoscopy is performed in both the diagnosis and treatment of GERD. In addition, endoscopic assessment of RE is useful to evaluate therapeutic effects. In the present review, the role of endoscopy in the management of GERD is summarized.

## DIAGNOSIS

### White light imaging

#### Reflux esophagitis

Mucosal breaks at the esophagogastric junction (EGJ) have been assessed in evaluating RE. There are many grading systems in use for evaluating the severity of RE, and now the Los Angeles (LA) classification is widely used.^7^ The circumferential extent of mucosal breaks is evaluated, and RE is classified into four grades (A–D). The LA classification has been validated^8^ and is significantly associated with esophageal acid exposure.^9^ Fair to moderate inter‐observer and intra‐observer agreements in the endoscopic assessment of RE using the classification are reported.^10^, ^11^ Recently, it has been reported that a deep‐learning model can increase the accuracy of interpretation of the severity of RE by inexperienced endoscopists.^12^

Mild erosive esophagitis, especially LA grade A, can be found in asymptomatic subjects.^13^, ^14^, ^15^ The recent consensus statements by experts (the Lyon consensus) concluded that severe erosive esophagitis (LA grades C and D), BE, and esophageal stricture are conclusive evidence for pathological reflux, but mild erosive esophagitis (LA grades A and B) is borderline or inconclusive evidence that should be confirmed by adjunctive supportive evidence.^16^

#### Minimal changes

**FIGURE 1:**
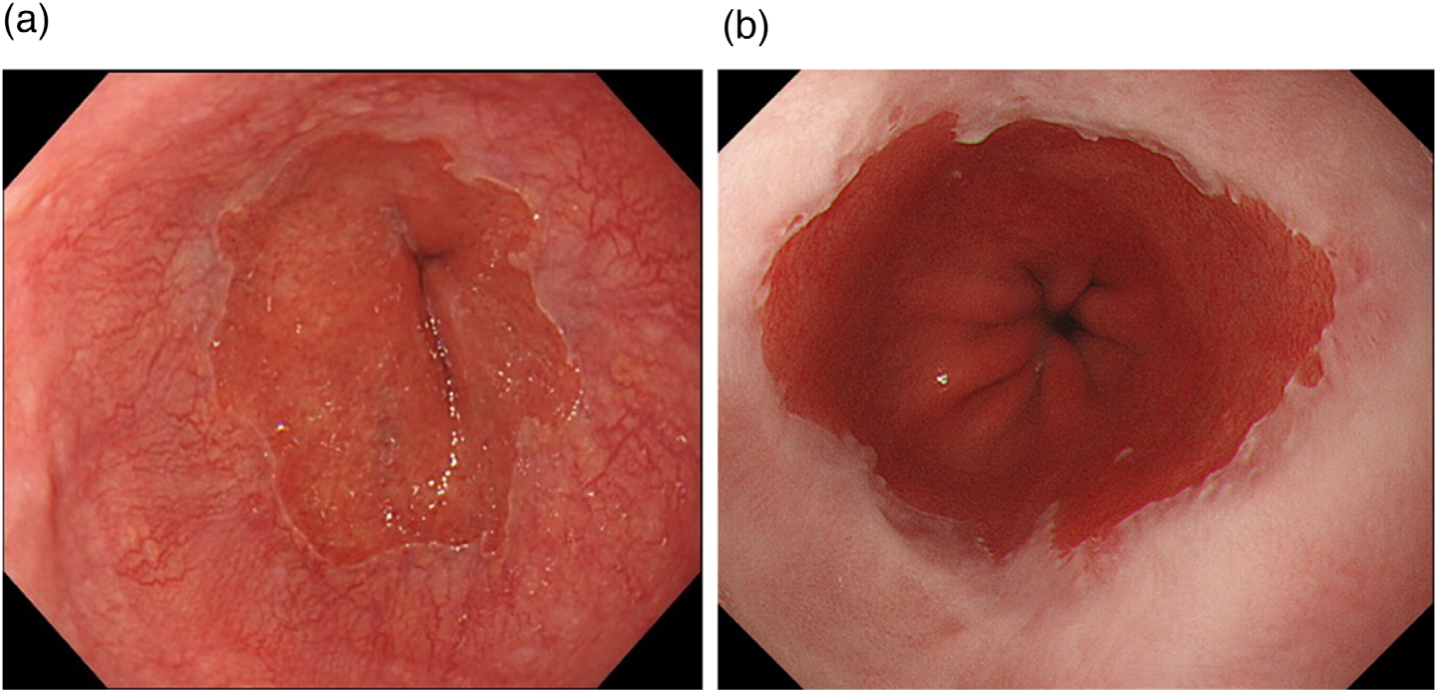
Grades N and M in the modified LA classification. (a) Palisade vessels can be observed circumferentially at the squamocolumnar junction (Grade N). (b) Whitish turbidity in the distal esophagus is observed, and palisade vessels cannot be observed (Grade M)

A modified LA classification with minimal changes (LA grade M) and normal mucosa (LA grade N) is accepted in Japan (Figure 1). LA grade M is defined as erythema without sharp demarcation, whitish turbidity, and/or invisibility of vessels.^17^, ^18^, ^19^ Magnifying endoscopies showed that minimal changes and histological findings related to gastroesophageal reflux were observed in patients with reflux symptoms more frequently than in those without reflux symptoms.^20^ It has been reported that the total number of acid reflux events detected by 24‐h esophageal pH monitoring was significantly higher than controls.^21^ Inter‐observer agreement among experienced endoscopists in the recognition of minimal changes was acceptable; inter‐observer agreement among inexperienced endoscopists was poor.^7^ A recent study showed that inter‐observer agreement in the endoscopic evaluation of LA grade M among Japanese endoscopists was also poor.^22^

#### Barrett's esophagus

Esophageal acid and/or bile acid exposure are related to the development of BE.^23^, ^24^, ^25^ A meta‐analysis showed that there was a significant association between GERD and BE, and the odds ratio was 2.90 (95% confidence interval [CI], 1.86–4.54).^26^ Intestinal‐type metaplasia is required in the United States,^27^ while BE can be diagnosed regardless of the presence of intestinal‐type metaplasia in Japan and the United Kingdom.^28^, ^29^ BE is categorized into two types based on the length of BE: short‐segment BE and long‐segment BE. An endoscopic grading system (The Prague C & M criteria) is used.^30^

### Histological assessments

Histological assessments are necessary to exclude eosinophilic esophagitis. In addition, papillary elongation (PE), basal cell hyperplasia (BCH), dilated intercellular spaces (DIS), intraepithelial inflammatory cells, and erosions may be related to GERD.^31^ These findings are validated,^32^ and a histological score using DIS, BCH and PE can be useful to distinguish between NERD and functional heartburn.^33^ However, GERD cannot be diagnosed only by histological findings.

### Chromoendoscopy

Since an inflamed mucosa does not contain glycogen, it shows unstained areas under Lugol chromoendoscopy. When histological findings were compared between stained and unstained areas in Lugol chromoendoscopy, Lugol unstained areas were more concordant with positive histological findings.^34^ Unstained streaks in Lugol chromoendoscopy have been reported as endoscopic findings for GERD.^35^ In this study unstained streaks were observed in 19 of 39 patients with NERD (49%), while they were only observed in one out of 38 controls (2.6%). In addition, typical pathological findings related to RE were observed in unstained areas more frequently than in stained areas. The relationship between this finding and GERD was confirmed in patients who improved their symptoms after the administration of antacids.^36^ Although Lugol chromoendoscopy is useful to detect an inflamed mucosa that could not be detected by white light imaging (WLI) in patients with NERD, Lugol solutions can cause chest pain, chest discomfort, and allergic reactions.^37^

### Magnification endoscopy

Findings in magnification endoscopy were seen more frequently in patients with NERD (69.2%) than in controls (20.5%).^20^ Endoscopic criteria for non‐erosive squamous mucosal injury by gastroesophageal reflux with high‐resolution magnification endoscopy were proposed: 1) Triangular indentations into the squamous mucosa by villiform columnar mucosa at the squamocolumnar junction (SCJ), 2) apical mucosal break at the vertex of a triangular indentation, 3) invisible palisade blood vessels in the squamous mucosa above the SCJ, 4) pinpoint or comma‐shaped blood vessels in squamous mucosa above the SCJ, 5) branching blood vessels in columnar mucosa below the SCJ, 6) “serrated SCJ” where more than three saw‐tooth incursions into the squamous mucosa with the depth of each saw‐tooth incursion greater or equal to its width are seen per radial gastric fold and 7) “villiform mucosa,” which is defined as villous−like mucosa immediately below the SCJ.^38^ The usefulness of these criteria was confirmed in patients with NERD.^39^ However, the inter‐observer agreement on these findings was quite low (kappa values, 0.18–0.28) except for invisible palisade blood vessels (kappa value, 0.59).

### Image‐enhanced endoscopy

#### Narrow‐band imaging

Narrow‐band imaging (NBI) is a digital technique in which blue light (390–445 nm) is used for the observation of microvascular patterns, and green light (530–550 nm) is used for the enhancement of contrast between superficial and deeper vessels in the mucosa. NBI can enhance visualization of the mucosal surface architecture and microvascular patterns. NBI is often used with magnification. Endoscopic features were identified with NBI in patients with GERD^40^: 1) Increased numbers, dilatation, and tortuosity of intrapapillary capillary loops (IPCLs), 2) presence of microerosions, 3) vascularity at the SCJ, 4) presence of columnar island in the distal esophagus, and 5) ridge‐villous pattern below the SCJ characterized by the presence of uniform, longitudinally aligned ridges alternating with a villiform pattern. Changes of IPCLs, microerosions, and increased vascularity at the SCJ among these findings were significant for detecting GERD. The sensitivity and specificity in changes of IPCLs were 60%–80%, and those in microerosions and increased vascularity at the SCJ were 40%–50% and 90%–100%, respectively. These findings were confirmed in another study.^41^ In addition, NBI can provide inter‐ and intra‐observer consistency in grading RE.^41^, ^42^

#### I‐scan

I‐scan is a new optical enhancement technique and software‐based real‐time modification of image sharpness, hue, and contrast that can provide high‐resolution images. It has been reported that an i‐scan could improve the identification of minimal changes in patients with NERD.^43^, ^44^ A cohort study showed that i‐scan could detect minimal changes more frequently in dyspeptic patients with GERD than in those patients without GERD or controls. Sensitivity, specificity, positive predictive value (PPV), and negative predictive value (NPV) of minimal changes detected by i‐scan in detecting GERD confirmed by the presence of RE or abnormal esophageal acid exposure time (AET) in 24‐h impedance‐pH monitoring were 51.35%, 67.33%, 36.54%, and 79.06%, respectively.^45^

#### Flexible spectral imaging color enhancement

Flexible spectral imaging color enhancement (FICE) is a software technology that uses post‐processing techniques to achieve improvement of visualization. A triangular indentation into the squamous mucosa that extended from the villiform columnar at the SCJ was proposed as a diagnostic finding for GERD in FICE. A pilot study showed that FICE could provide higher sensitivity, NPV, and accuracy than WLI.^46^ Sensitivity, specificity, PPV, NPV and accuracy of FICE were 77.8%, 83.3%, 93.3%, 55.6%, and 79.2%, respectively. However, the inter‐observer agreement was poor.

#### Blue laser imaging and linked color imaging

Blue laser imaging (BLI) and linked color imaging (LCI) are image‐enhanced endoscopy (IEE) technologies. Blue and green color information and red color information are corrected separately. Similar to NBI, BLI uses blue and green color information, while LCI uses the information of all three colors and enhances color differences. It has been reported that LCI can improve the detection of minimal changes in patients with NERD.^47^ Several studies were conducted to compare the detection of GERD between BLI, LCI, and WLI. Takeda et al. reported that LCI can improve the visibility of RE.^48^ However, Lee et al. reported that inter‐observer agreements in diagnosing RE, including minimal changes in BLI and LCI, were not high.^49^

### Confocal laser endomicroscopy

Confocal laser endomicroscopy (CLE) can provide surface and subsurface imaging with magnification and up to 250μm below the tissue surface. Since PE is a typical histological feature of GERD, measuring surface to papillary tip (S‐P) distance could differentiate between inflamed and normal mucosa. The S‐P distance measured with CLE was correlated to histological assessment, and the distance in patients with RE was significantly shorter than that in controls.^50^ Increased IPCLs and DIS were also observed with CLE in patients with NERD.^51^ Although a dedicated confocal endomicroscope was used in these studies, it is no longer commercially available. Recently, the probe‐based CLE (pCLE) became commercially available, and it can provide CLE imaging 55–65 μm below the tissue surface in vivo during endoscopy. A recent study evaluated esophageal epithelial barrier function (EBF) using pCLE; however, pCLE was not able to differentiate between GERD and non‐GERD and did not correlate with EBF evaluated in vitro.^52^

### Mucosal impedance testing

Esophageal mucosal exposure to injurious agents could lead to mucosal structural changes such as DIS. Mucosal impedance testing (MIT) has been performed to assess esophageal mucosa integrity.^53^ it showed that increased DIS correlated with lower MIT values.^54^, ^55^ An impedance measurement probe has been developed, which allows measuring MIT values during endoscopy.^56^, ^57^ Studies with this probe have shown that MIT can discriminate between GERD and non‐GERD, and lower MIT values were observed in patients with GERD than in those without GERD.^56^, ^57^, ^58^, ^59^, ^60^ A cut‐off value of 2019 Ω at 5 cm above the SCJ was proposed to diagnose objective GERD with a sensitivity of 76% and specificity of 95%.^58^ A newly designed balloon mucosal impedance catheter has been developed. MIT with the balloon catheter allows endoscopists to differentiate GERD and non‐GERD instantly during endoscopy.^61^

Mucosal admittance measurement has been developed to measure mucosal integrity. Mucosal admittance in patients with GERD was significantly higher than in those with functional heartburn.^62^ In addition, mucosal admittance was negatively correlated with baseline impedance and positively correlated with AET measured by esophageal impedance‐pH monitoring. Mucosal admittance measurement with histological assessment revealed that mucosal admittance was more closely correlated with BCH than DIS.^63^

### Risk stratifications of GERD

Hiatal hernia is an important risk factor for GERD. Since there is a barrier function against gastroesophageal reflux at the EGJ, weakening of this barrier function could lead to the development of GERD. The gastroesophageal flap valve (GEFV) is graded based on endoscopic features of the EGJ, and it was reported that GEFV grade III or IV was significantly associated with the development of RE.^64^, ^65^

### Surveillance of BE

Since BE is a premalignant condition, it is important to perform screening and surveillance of BE by endoscopy. A systematic review and meta‐analysis showed that endoscopic surveillance in patients with BE was associated with the detection of earlier‐stage esophageal adenocarcinoma.^66^ Thus, surveillance endoscopy is recommended in the American Society for Gastrointestinal Endoscopy (ASGE) guidelines.^67^ WLI with random biopsies with Seattle protocol has been recommended during the endoscopic surveillance in patients with BE. Recently, chromoendoscopy or IEEs have been developed, and the usefulness of these techniques with target biopsy has been reported. Based on the ASGE preservation and incorporation of valuable endoscopic innovations (PIVI) on imaging technology, an imaging technology required sensitivity of 90% or greater and an NPV of 98% or greater for detecting high‐grade dysplasia or esophageal adenocarcinoma to eliminate random biopsies during surveillance of BE.^68^ In addition, the new technology should have a sufficiently high (80%) sensitivity. A systematic review and meta‐analysis indicated that target biopsies with acetic acid chromoendoscopy, NBI, and endoscope‐based CLE met the ASGE PIVI thresholds when endoscopists with expertise in advanced imaging techniques use these techniques.^69^ The latest ASGE guideline on screening and surveillance of BE recommends the use of chromoendoscopy or virtual chromoendoscopy, such as NBI, in addition to WLI during surveillance of BE; however, it still recommends random biopsies with Seattle protocol.^67^

## TREATMENTS

Several endoscopic treatments have been proposed for patients with GERD. Since proton pump inhibitor (PPI) or vonoprazan (VPZ), which is available in several Asian countries, is the first choice for the treatment of GERD; the endoscopic treatments are performed in patients with PPI‐ or VPZ‐refractory GERD. Endoscopic radiofrequency ablation (RFA), endoscopic fundoplication, and endoscopic mucosal resection are currently performed (Table 1). Other techniques, such as injection of bulking agents and endoscopic suturing had been performed; however, they are no longer performed due to poor efficacy or safety concerns.^70^

**TABLE 1:** Endoscopic treatments for gastroesophageal reflux disease (GERD)

**Method**	**Treatment name [device]**
Radiofrequency ablation	Stretta
Endoscopic fundoplication	Transoral incisionless fundoplication [EndophyX]
	Endoscopic full‐thickness plication [GERDx]
	Medigus ultrasonic surgical endostapler [MUSE]
Endoscopic mucosal resection	Anti‐reflux mucosectomy (ARMS)
	Endoscopic submucosal resection for GERD (ESD‐G)
	Endoscopic band ligation
	Peroral endoscopic cardial constrction
	Resection and plication (RAP) [OverStitch]

### Radiofrequency ablation

The Stretta system (Mederi Therapeutics, Norwalk, CT, USA) applies radiofrequency energy to the muscles of the EGJ and gastric cardia. A four‐needle balloon catheter is used with rotation and linear movements to deliver radiofrequency energy to multiple sites. Several mechanisms of action of the Stretta have been suggested: increased gastric yield pressure, hypertrophy of muscularis propria at the EGJ, decreased EGJ compliance,^71^ and inhibited triggering of transient lower esophageal sphincter relaxation.^72^, ^73^

Several cohort studies and randomized controlled trials (RCTs) have shown the efficacy of the Stretta in the treatment of GERD. The long‐term efficacy (10 years) was evaluated in 217 patients with medically refractory GERD; normalization of GERD‐health‐related quality of life (GERD‐HRQL) was achieved in 72% of patients.^74^ In addition, 41% of patients could eliminate PPI use, and a 60% or greater increase in satisfaction occurred in 54% of patients. A systematic review and meta‐analysis including 1441 patients in 18 studies showed a significant improvement in GERD‐HRQL. Moreover, a DeMeester score indicating AET significantly decreased from 44.4 to 28.5.^75^ A subsequent systematic review and meta‐analysis including 2468 patients in 28 studies (four RCTs, 23 cohort studies, and one registry) confirmed the efficacy of the Stretta.^76^ Adverse events with the Stretta are chest pain, transient fever, and esophageal ulcers, but these adverse events are usually mild.^70^ The guidelines by the Society of American Gastrointestinal and Endoscopic Surgeons advocate the use of RFA in selected patients with GERD.^77^ Nevertheless, another study showed conflicting results,^78^ so the efficacy of the Stretta must be confirmed.

### Endoscopic fundoplication

#### Transoral incisionless fundoplication

The EsophyX (EndoGastric Solutions, Redmond, WA, USA) is available as a fundoplication device. It can reduce a hiatal hernia and create a valve 2 to 4 cm in length and a greater than 270° circumferential wrap.

Several RCTs and systemic reviews have shown the efficacy of transoral incisionless fundoplication (TIF). A systematic review and meta‐analysis including 963 patients in 18 studies (five RCTs and 13 prospective observational studies) showed that the pooled relative risk for response to TIF versus PPIs/sham was 2.44 (95% CI, 1.25–4.79).^79^ Although the total number of refluxes was reduced after TIF compared with the PPIs/sham group, the ACT and the number of acid refluxes did not significantly decrease. Factors predicting good outcomes with TIF were pre‐procedure GEFV grades I–II, no hiatal hernia or hernia less than 2 cm, absence of ineffective esophageal motility, the number of fasteners deployed, age more than 50 years, and persistence of symptoms (GERD‐HRQL more than 15 on PPIs).^80^, ^81^ Several severe adverse events including perforation, pneumothorax, and bleeding were reported although these events were rare.^82^

#### Endoscopic full‐thickness plication

Endoscopic full‐thickness plication (EFTP) was performed with the Plicator device (Ethicon Endosurgery, Somerville, NJ, USA); however, this device is no longer commercially available. Recently, the GERDx system (G‐SURG GmbH, Seeon‐Seebruck, Germany) has become available as a new EFTP device. A single suture was initially performed below the EGJ; however, this method could not create an effective anti‐reflux barrier.^83^ Subsequently, multiple sutures were placed to create a robust anti‐reflux valve.

A prospective study including 36 patients showed improvement of symptoms in 92% of patients, and 89% of patients could eliminate PPI use at 1‐year follow‐up.^84^ A significant reduction of AET was achieved. A multicenter study including 41 patients confirmed these results.^85^, ^86^ Recently, an RCT including 70 patients reported^87^ that more than 50% improvement in the GERD‐HRQL score at 3 months was more frequently achieved in the EFTP group than the sham therapy group (65.7% vs. 2.9%). In the EFTP group, 62.8% of patients could eliminate PPI use at 12 months after the procedure, while only 11.4% in the sham group could eliminate PPI use. pH parameters partially improved at 3 months, but not at 12 months. Adverse events with the GERDx were pain in the abdomen, shoulder, and chest. These adverse events were minor, and no long‐term adverse event was reported.

#### Medigus ultrasonic surgical endostapler

The medigus ultrasonic surgical endostapler (Medigus, Omer, Israel) is an endoscopic stapling device. The camera along with the light source allows for direct visualization of the staple site selection, and the ultrasonic range finder helps in assessing the tissue thickness before firing the staples.^88^

A multicenter prospective trial including 66 patients with 6 months follow‐up showed that improvement of GERD‐HRQL was achieved in 73% of patients, and 64.6% of patients could discontinue PPI use.^89^ AET significantly decreased 6 months after the procedure. Long‐term outcomes up to 4 years were also reported. The proportion of patients who remained off daily PPI use were 83.8% at 6 months and 69.4% at 4 years after the procedure. HRQL scores were significantly decreased from baseline to 6 months and 4 years post‐procedure.^90^

### Endoscopic mucosal resection

#### Anti‐reflux mucosectomy

Anti‐reflux mucosectomy (ARMS) creates mucosal defects that lead to scarring during the healing process, which causes narrowing of the EGJ opening. Mucosal resection is performed in approximately two‐thirds or four‐fifths of the circumferential on the lesser curvature mucosa of the cardia. In a retroflex view from the stomach, the mucosal defect appears as a butterfly shape. Originally, ARMS was performed by endoscopic submucosal dissection (ESD). Now, endoscopic mucosal resection with cap (EMR‐C) or band‐technique EMR (EMR‐L) is used.

Circumferential mucosal resection was performed in patients with short‐segment BE with high‐grade dysplasia, which made significant improvement of the patient's symptoms related to GERD.^91^ ARMS was developed based on this experience. A pilot study including 10 patients showed significant improvements of the DeMeester scores, AET (29.1%–3.1%), and fraction time absorbance more than >0.14 of bile reflux (52%–4%). All patients could discontinue PPI use.^92^ A subsequent study including 19 patients showed by EMR‐L techniques that two‐thirds of patients obtained symptomatic improvement and were able to discontinue their PPI.^93^ A study comparing ARMS and laparoscopic Nissen fundoplication (NF) in 33 patients showed that ARMS groups had significantly shorter operation time, less estimated blood loss, shorter hospital stay, less pain at discharge, earlier narcotic discontinuation, and earlier return to activities of daily living.^94^ GERD‐HRQL and dysphagia scores were comparable between ARMS and NF. Recently, a study including 109 patients with 3 years of follow‐up showed significant improvement of both symptoms and reflux parameters (AET and DeMeester score).^95^

#### ESD for GERD

ESD performed at the EGJ (ESD‐G) was reported.^96^ Differences between ARMS and ESD‐G were related to the resection approach and the width of the mucosal defect. ARMS was performed with a retroflex view from the stomach, while ESD‐G was performed with an anterograde view from the stomach. The range of mucosal resection in ESD‐G was limited to half of the circumference of the EGJ lumen. The study included 13 patients of whom 12 patients had significant improvement of symptoms; however, only three patients could discontinue PPI use.

#### Other techniques

Endoscopic band ligation had been reported and several bands were applied at the EGJ.^97^ The study, including 150 patients, showed significant improvement of GERD‐HRQL and RE. Mild dysphagia and epigastric pain were reported as adverse events.

A new endoscopic technique in gastric constriction in GERD (peroral endoscopic cardial constriction) was reported.^98^ The study, including 13 patients, showed significant improvement of GERD‐HRQL and AET.

A technique that involves partial mucosal resection followed by plication with the OverStitch device (Apollo Endosurgery) was reported (resection and plication). A pilot study, including 10 patients, showed significant improvement of GERD‐HRQL and 80% of elimination of PPI use.^99^

## CONCLUSION

Endoscopy is useful not only in diagnosis but also in risk stratification and treatment in the management of GERD.

## CONFLICT OF INTEREST

The authors declare no conflict of interest for this article. Toshio Uraoka is a Deputy Editor‐in‐Chief of DEN Open.

## FUNDING INFORMATION

This work was supported by the Japan Society for the Promotion of Science (JSPS) KAKENHI for early‐career scientists [JP18K15772 to Shiko Kuribayashi].
